# The Editor's Letter-Box

**Published:** 1915-10-16

**Authors:** 


*To the Editor of* The Hospital.

*To the Editor of* The Hospital.

Sir,—In perusing this week's issue of The Hospital, I notice the advertisement for secretary and house steward at the Norfolk and Norwich Hospital, at the meagre salary of £250 per annum, plus board and lodging.

Surely the committee cannot expect a highly qualified and experienced man at that paltry figure. Of course there is the addition of board and lodging. The lodging, which may only consist of bed and sitting room usually given to house surgeons, would not—in that case—be much inducement for a married man to apply, as most of the candidates would be, according to the announcement of age limit, thirty to fifty years.

I think most hospital officials will agree that the remuneration for secretarial posts does not improve. The work is becoming more onerous, especially so to-day with those institutions that are treating military patients.

We are now, like everyone else, heavily taxed, which we bear patiently, without much hope of saving for old age, and no set scheme of pensions as yet afoot to look forward to.

Perhaps some future generation of hospital officer will be treated more worthily of his hire.

I enclose my card, and remain yours faithfully,

October 8, 1915. Hospital Officer.

*** We refrain this week from commenting on the important issues raised by the above letters on the vacancy at Norwich and the advertisement. We hope that, through some misapprehension, a mistake has been made by the authorities in regard to the advertisement in question, which may lead to its being withdrawn that an amended announcement may be substituted.—Ed. The Hospital.

